# Prediction of survival and analysis of prognostic factors for patients with AFP negative hepatocellular carcinoma: a population-based study

**DOI:** 10.1186/s12876-024-03185-z

**Published:** 2024-03-04

**Authors:** Chengyu Liu, Zikang Li, Zhilei Zhang, Jinlong Li, Congxi Xu, Yuming Jia, Chong Zhang, Wuhan Yang, Wenchuan Wang, Xiaojuan Wang, Kuopeng Liang, Li Peng, Jitao Wang

**Affiliations:** 1 Graduate School of Hebei Medical University, Shijiazhuang, Hebei China; 2 Hepatobiliary Surgery Department of the Fourth Hospital of Hebei Medical University, 169 Tianshan Street, Shijiazhuang, Hebei China; 3 Xingtai Key Laboratory of Precision Medicine for Liver Cirrhosis and Portal Hypertension, Xingtai People’s Hospital of Hebei Medical University, Xingtai, Hebei China; 4 Hebei Provincial Key Laboratory of Cirrhosis & Portal Hypertension, 145 Xinhua North Road, Xingtai, Hebei China

**Keywords:** Hepatocellular Carcinoma, Alpha-fetoprotein, Nomogram, Predictive model

## Abstract

**Purpose:**

Hepatocellular carcinoma (HCC) has a poor prognosis, and alpha-fetoprotein (AFP) is widely used to evaluate HCC. However, the proportion of AFP-negative individuals cannot be disregarded. This study aimed to establish a nomogram of risk factors affecting the prognosis of patients with AFP-negative HCC and to evaluate its diagnostic efficiency.

**Patients and methods:**

Data from patients with AFP-negative initial diagnosis of HCC (ANHC) between 2004 and 2015 were collected from the Surveillance, Epidemiology, and End Results database for model establishment and validation. We randomly divided overall cohort into the training or validation cohort (7:3). Univariate and multivariate Cox regression analysis were used to identify the risk factors. We constructed nomograms with overall survival (OS) and cancer-specific survival (CSS) as clinical endpoint events and constructed survival analysis by using Kaplan-Meier curve. Also, we conducted internal validation with Receiver Operating Characteristic (ROC) analysis and Decision curve analysis (DCA) to validate the clinical value of the model.

**Results:**

This study included 1811 patients (1409 men; 64.7% were Caucasian; the average age was 64 years; 60.7% were married). In the multivariate analysis, the independent risk factors affecting prognosis were age, ethnicity, year of diagnosis, tumor size, tumor grade, surgery, chemotherapy, and radiotherapy. The nomogram-based model related C-indexes were 0.762 (95% confidence interval (CI): 0.752–0.772) and 0.752 (95% CI: 0.740–0.769) for predicting OS, and 0.785 (95% CI: 0.774–0.795) and 0.779 (95% CI: 0.762–0.795) for predicting CSS. The nomogram model showed that the predicted death was consistent with the actual value. The ROC analysis and DCA showed that the nomogram had good clinical value compared with TNM staging.

**Conclusion:**

The age(HR:1.012, 95% CI: 1.006–1.018, *P*-value < 0.001), ethnicity(African-American: HR:0.946, 95% CI: 0.783–1.212, *P*-value: 0.66; Others: HR:0.737, 95% CI: 0.613–0.887, *P*-value: 0.001), tumor diameter(HR:1.006, 95% CI: 1.004–1.008, *P*-value < 0.001), year of diagnosis (HR:0.852, 95% CI: 0.729–0.997, *P*-value: 0.046), tumor grade(Grade 2: HR:1.124, 95% CI: 0.953–1.326, *P*-value: 0.164; Grade 3: HR:1.984, 95% CI: 1.574–2.501, *P*-value < 0.001; Grade 4: HR:2.119, 95% CI: 1.115–4.027, *P*-value: 0.022), surgery(Liver Resection: HR:0.193, 95% CI: 0.160–0.234, *P*-value < 0.001; Liver Transplant: HR:0.102, 95% CI: 0.072–0.145, *P*-value < 0.001), chemotherapy(HR:0.561, 95% CI: 0.471–0.668, *P*-value < 0.001), and radiotherapy(HR:0.641, 95% CI: 0.463–0.887, *P*-value:0.007) were independent prognostic factors for patients with ANHC. We developed a nomogram model for predicting the OS and CSS of patients with ANHC, with a good predictive performance.

**Supplementary Information:**

The online version contains supplementary material available at 10.1186/s12876-024-03185-z.

## Introduction

Hepatocellular carcinoma (HCC) is a major end-stage liver disease with the sixth highest incidence rate and third highest cause of cancer-related mortality [1, 2]. According to global cancer statistics for 2020, there were approximately 906,000 new cases and 830,000 HCC deaths. Early and accurate diagnosis and treatment are crucial for mitigating these threats.

Alpha-fetoprotein (AFP) is currently the most widely used biomarker for the diagnosis, monitoring, evaluation of prognosis, and treatment of HCC [2–6]. The elevation of tumor markers, mainly AFP, is independently associated with poor prognostic features, such as shortened survival [7–11]. However, in the HCC patient population, AFP-negative hepatocellular carcinoma (ANHC), i.e., AFP < 20 ng/mL at initial diagnosis, is an important type that causes many patients to lose early diagnosis and treatment and accounts for approximately 30-40% of patients [12]. Therefore, the survival and prognosis of ANHC patients are still a matter of concern. Unfortunately, there are relatively few prognostic studies based on the ANHC cohort [9, 13, 14].

We analyzed the impact of the patient’s general condition, tumor indications, and treatment methods on survival, identified relevant risk factors, and constructed a nomogram to guide prognostic evaluation and early diagnosis of ANHC.

## Materials and methods

### Research design and patients

This study collected the clinical data of patients with ANHC from the Surveillance, Epidemiology, and End Results (SEER) database. The inclusion criteria for this study were patients diagnosed between 2004 and 2015 with a primary tumor in the liver, and the third edition of the International Classification of Tumor Diseases was 8170/3: hepatocellular carcinoma, NOS. The confirmed cases included in this study required HCC to be confirmed and diagnosed through pathological examination and were initially diagnosed as AFP-negative (< 20 ng/mL). The exclusion criteria were AFP positivity (> 20 ng/mL) or unknown status at initial diagnosis, unknown tumor size, unknown marital status at diagnosis, unknown surgical treatment, and lack of complete survival time.

### Data collection and statistical variable definition

The following clinical information was collected for further analysis: baseline demographic data, including age, ethnicity, gender, year of diagnosis, marital status, survival time, cancer-specific survival rate, and survival status; tumor characteristics, including tumor size (maximum diameter, in cm), pathological tumor grading; and TNM stage according to the American Joint Committee on Cancer 6th TNM stage; and treatment strategies, including surgery (liver resection and liver transplantation), chemotherapy, and radiotherapy at the primary site.

Gender was categorized as male or female. The year of diagnosis was categorized according to before and after 2009. Ethnicity was categorized into three racial groups: Caucasians, African Americans, and others. Marital status at the time of diagnosis was divided into married and unmarried/divorced/widowed groups. Surgery types were divided into no surgery, liver resection, and liver transplantation. For radiotherapy and chemotherapy, patients were classified as having, not having, or unknown.

### Statistical analysis

We randomly divided all eligible patients with ANHC into two groups at 7:3: training cohort (*n* = 1267) and validation cohort (*n* = 544). Univariate COX analysis was conducted in the training cohort with overall survival (OS) as the endpoint, and statistically significant individuals (*p* < 0.1) were selected for the multivariate COX analysis. Furthermore, a nomogram was constructed using OS and cancer-specific survival (CSS) as endpoints (*P* < 0.05). The two nomogram groups were validated using a validation cohort. A nomogram model for independent prognostic factors was established based on multivariate Cox regression analysis (*p* < 0.05), and the fit was evaluated using a consistency index (C-index).

In the training and validation cohorts, calibration curves for the first, third, and fifth years were established based on the nomogram model by comparing the predicted and actual observed values of the nomogram in the OS and CSS groups. Calibration curves of the validation cohort were established in the same way. The Receiver Operating Characteristic (ROC) table and Decision Curve Analysis (DCA) evaluation model was fitted, under which diagnostic efficacy was compared with that of TNM staging. Clinical information extraction was performed using SEER*Stat software version 8.3.8 (www.seer.cancer.gov/seerstat). Data analysis was carried out using R software version 4.2.2(R-Project, https://www.r-project.org/). A *P*-value < 0.05 was considered statistically significant.

## Results

### Patient characteristics

Based on the selection criteria, 1811 patients from the SEER database diagnosed with ANHC (1409 men; average age, 64 years; age range 13–96 years) were selected for inclusion in this study. 1054 (58.6%) patients were diagnosed after 2009. The most common ethnicity was Caucasian, accounting for 64.7% of the study population. In total, 1099 patients (60.7%) were married. In terms of tumor characteristics, the median tumor diameter was 4.5 cm (IQR, 2.6–7.5 cm). Pathological grades I and II were observed in a total of 1584 individuals, representing 87.5% of the total population. TNM stages I and II were identified in 1350 individuals, accounting for 74.6% of the total. In terms of treatment, the majority of patients (1075, 59.4%) received surgical treatment, while liver transplantation was performed in 259 patients (14.3%). Chemotherapy was administered to 560 patients (30.9%) and 114 patients (6.3%) received radiotherapy. 70% of the patients were allocated to the training cohort and 30% to the validation cohort. The baseline characteristics of the total, training, and validation cohorts are shown in Table 1.

**Table 1:** Baseline data of patients with combined ANHC about demographic and clinical characteristics of tumor and therapy

Variables	Total Cohort	Training Cohort	Validation Cohort
All Patient	1811	1267	544
Age at diagnosis			
<55	355(19.6)^a^	237(18.7)	118(21.7)
≥55	1456(80.4)	1030(81.3)	426(78.3)
Ethnicity			
Caucasian	1171(64.7)	821(64.8)	350(64.3)
African–American	167(9.2)	124(9.8)	43(7.9)
Others	473(26.1)	322(25.4)	151(27.8)
Gender			
Male	1409(77.8)	980(77.3)	429(78.9)
Female	402(22.2)	287(22.7)	115(21.1)
Year of Diagnosis			
After 2009	1054(58.6)	729(57.5)	325(59.7)
Before 2009	757(41.4)	538(42.5)	219(40.3)
Grade			
Grade I	731(40.4)	506(39.9)	225(41.4)
Grade II	853(47.1)	597(47.1)	256(47.1)
Grade III	210(11.6)	151(11.9)	59(10.8)
Grade IV	17(0.9)	13 [1]	4(0.7)
TNM Stage			
I	992(54.8)	681(53.7)	311(57.2)
II	358(19.8)	262(20.7)	96(17.6)
III	328(18.1)	228 [18]	100(18.4)
IV	133(7.3)	96(7.6)	37(6.8)
Surgery			
No surgery	736(40.6)	521(41.1)	215(39.5)
Liver Resection	816(45.1)	566(44.7)	250(46)
Liver Transplant	259(14.3)	180(14.2)	79(14.5)
Chemotherapy			
No/Unknown	1251(69.1)	870(68.7)	381(70)
Yes	560(30.9)	397(31.3)	163 [30]
Radiation			
No/unknown	1697(93.7)	1194(94.2)	503(92.5)
Yes	114(6.3)	73(5.8)	41(7.5)
Marital Group			
Married	1099(60.7)	761(60.1)	338(62.1)
Single/divorced/widowed	712(39.3)	506(39.9)	206(37.9)
Tumor Size			
<5 cm	1011(55.8)	702(55.4)	309(56.8)
≥5 cm	800(44.2)	565(44.6)	235(43.2)

^a^ The numbers outside and in every parentheses correspond to the number of patients (n) and the percentage of the respective group (%)

### Univariate and Multivariate Analysis for OS

In the total cohort, the median OS was 38.0 months (95% CI:33.6–42.4 months), with 1-year, 3-year, and 5-year OS rates of 73.8%, 50.9%, and 39.3%, respectively. We analyzed risk factors affecting the early prognosis of patients with ANHC through univariate and multivariate COX regression analysis. In the analysis of overall survival, univariate COX regression analysis showed that age, marital status, ethnicity, year of diagnosis, tumor pathological grade, tumor diameter, surgery, chemotherapy, and radiotherapy were significant indicators affecting the overall survival of these patients. Further multivariate COX regression analysis was performed on significant individuals with independent predictors, including age(HR:1.012, 95% CI: 1.006–1.018, *P*-value < 0.001), ethnicity(African-American: HR:0.946, 95% CI: 0.783–1.212, *P*-value: 0.66; Others: HR:0.737, 95% CI: 0.613–0.887, *P*-value: 0.001), tumor diameter(HR:1.006, 95% CI: 1.004–1.008, *P*-value < 0.001), year of diagnosis (HR:0.852, 95% CI: 0.729–0.997, *P*-value: 0.046), tumor grade(Grade 2: HR:1.124, 95% CI: 0.953–1.326, *P*-value: 0.164; Grade 3: HR:1.984, 95% CI: 1.574–2.501, *P*-value < 0.001; Grade 4: HR:2.119, 95% CI: 1.115–4.027, *P*-value: 0.022), surgery(Liver Resection: HR:0.193, 95% CI: 0.160–0.234, *P*-value < 0.001; Liver Transplant: HR:0.102, 95% CI: 0.072–0.145, *P*-value < 0.001), chemotherapy(HR:0.561, 95% CI: 0.471–0.668, *P*-value < 0.001), and radiotherapy(HR:0.641, 95% CI: 0.463–0.887, *P*-value:0.007). The hazard ratios and *P*-values are presented in Table 2.

**Table 2:** Univariate analysis and multivariate analysis of factors of overall survival (OS) of ANHC.

Variables	Categories	Univariate analysis				Multivariate analysis		
		HR	95% CI	*P* Value		HR	95% CI	*P* Value
Age at Diagnosis		1.026	1.02–1.033	< 0.001		1.012	1.006–1.018	< 0.001
Tumor Size		1.007	1.006–1.009	< 0.001		1.006	1.004–1.008	< 0.001
Ethnicity	African–American	1.095	0.858–1.397	0.467		0.946	0.738–1.212	0.66
	Others	0.767	0.64–0.919	0.004		0.737	0.613–0.887	0.001
Year of Diagnosis		0.852	0.729–0.997	0.046		0.807	0.688–0.948	0.009
Gender		0.996	0.836–1.188	0.967		NA	NA	NA
Grade	Grade 2	0.948	0.806–1.115	0.518		1.124	0.953–1.326	0.164
	Grade 3	1.586	1.263–1.99	< 0.001		1.984	1.574–2.501	< 0.001
	Grade 4	2.518	1.338–4.735	0.004		2.119	1.115–4.027	0.022
Surgery	LR	0.253	0.215–0.297	< 0.001		0.193	0.16–0.234	< 0.001
	LT	0.1	0.073–0.138	< 0.001		0.102	0.072–0.145	< 0.001
Chemotherapy		1.217	1.041–1.423	0.014		0.561	0.471–0.668	< 0.001
Radiation		1.445	1.057–1.977	0.021		0.641	0.463–0.887	0.007
Marital Group		0.767	0.66–0.891	0.001		0.914	0.782–1.069	0.262

Abbreviations: HR: Hazard Ratio, CI: Confidence interval, LR: Liver Resection, LT: Liver Transplant

### Nomogram for Predicting OS

**Fig. 1:**
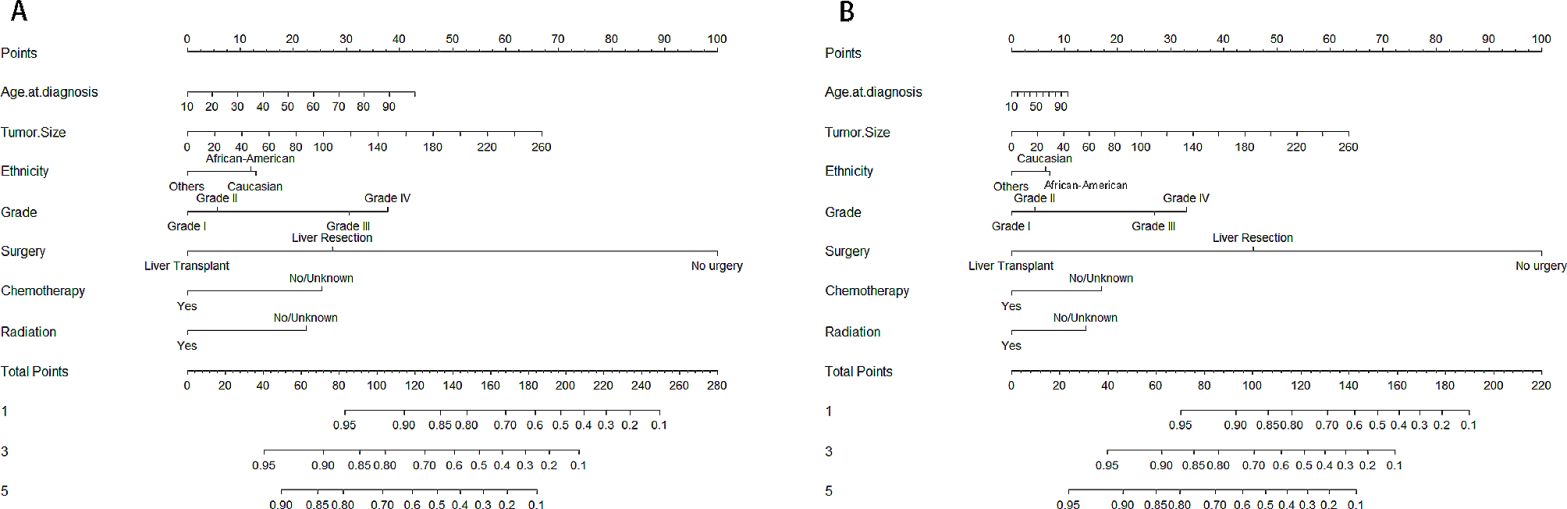
In the training cohort, the nomogram models for predicting the overall survival (A) and cancer-specific survival (B) for 1, 3, and 5 years of ANHC was constructed by combining age, tumor diameter, ethnicity, pathological grade, surgical, chemotherapy, and radiotherapy conditions

A prognostic evaluation of the nomogram for predicting OS in patients with ANHC has been developed and validated based on the independent prognostic variables (age, tumor diameter, ethnicity, pathological grade, surgical, chemotherapy, and radiotherapy conditions) identified in the multivariate analysis (Fig. 1A). Considering that the year of diagnosis has no clinical significance as a predictor variable, we did not include it in the nomogram. In the training and validation cohorts, the nomogram showed satisfactory performance in predicting OS with C-index of 0.762 (95% CI: 0.752–0.772) and 0.752 (95% CI: 0.740–0.769), respectively. In the training and validation cohorts, the calibration curves of 1-year, 3-year and 5-year OS probabilities showed the best consistency between the actual observations and the model predictions based on the nomogram (Supplementary Fig. 1).

**Fig. 2:**
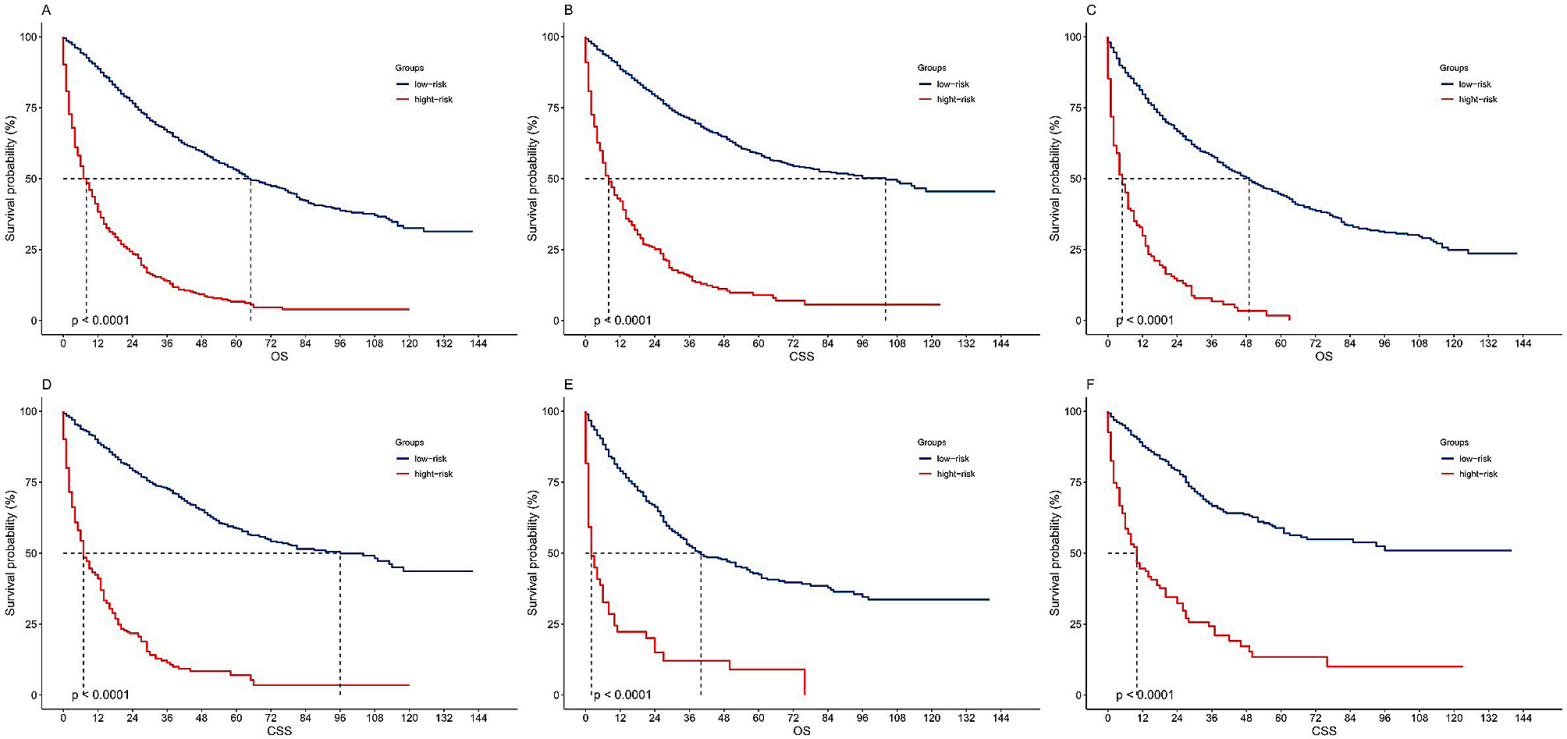
Kaplan–Meier curves for risk classification based on the nomogram scores. (A) In all cohort of OS group; (B) In all cohort of CSS group; (C) In the training cohort of OS group; (D) In the training cohort of CSS group; (E) In the validation cohort of OS group; (F) In the validation cohort of CSS group

By applying the best cutoff value of the nomogram in the training cohort, we developed risk stratification for OS. According to the nomogram-based model score, the whole patients with ANHC were divided into low-risk group (≤ 177.6 score) and high-risk group (> 177.6 score). Kaplan-Meier analysis showed that the median OS of the low-risk and high-risk groups were 65 months (95% CI: 60–70 months) and 8 months (7–10 months), respectively (*P* < 0.001, Fig. 2A). In the training cohort, the median OS values of the low-risk group and the high-risk group were 45 months (95% CI:43–55 months) and 5 months (4–7 months), respectively (*P* < 0.001, Fig. 2C). In the validation cohort, the median OS values of the low-risk and high-risk groups were 40 months (95% CI:35–56 months) and 2 months (1–6 months), respectively (*P* < 0.001, Fig. 2E).

### Nomogram for predicting CSS

The nomogram for predicting CSS in patients with ANHC has also been developed and validated based on all independent prognostic variables identified in the multivariate analysis (Fig. 1B). The C-index was 0.785 (95% CI: 0.774–0.795) and 0.779 (95% CI: 0.762–0.795) in the training and validation cohorts, respectively. The calibration curves of 1-year, 3-year and 5-year CSS was also been formed (Supplementary Fig. 2).

By applying the same method, all ANHC patients were divided into low-risk group (≤ 145.4 score) and high-risk group (> 145.4 score) under the condition of setting CSS as terminal event. Kaplan-Meier analysis showed that the median CSS of the low-risk and high-risk groups were 104 months (95% CI: 79–128 months) and 8 months (7–10 months), respectively (*P* < 0.001, Fig. 2B). In the training cohort, the median CSS values of the low-risk group and the high-risk group were 96 months (95% CI: 74–106 months) and 7 months (6–10 months), respectively (*P* < 0.001, Fig. 2D). In the validation cohort, the median CSS values of the low-risk and high-risk groups were 105 months (95% CI:67–114 months) and 10 months (7–17 months), respectively (*P* < 0.001, Fig. 2F).

## Discussion

Based on SEER database, our study collected the largest cohort of AFP-negative patients available and constructed a new nomogram through data analysis. Compared with traditional TNM staging, it has improved effectiveness in evaluating patient prognosis. The independent risk factors included in our final nomogram were age, tumor diameter, ethnicity, pathological grade, surgical, chemotherapy, and radiotherapy conditions.

Patients with HCC constitute a substantial population characterized by a poor prognosis and a relatively short survival period. The 2022 Barcelona Clinic Liver Cancer guidelines propose that AFP is an important indicator that affects the treatment, prognosis, and clinical decision-making for HCC [15]. The concentration of AFP in adults is about 5–10 ng/mL, and the commonly used optimal screening threshold is 16–20 ng/mL [6, 7, 16, 17]. Nearly half of HCC patients are AFP-negative, most of which are early and small HCC; in advanced patients, 15-30% of serum have AFP values at the normal range (< 20 ng/mL) [18, 19]. Therefore, the ANHC population should be non-negligible.

Usually, OS, with all-cause mortality as the endpoint, is widely used in clinical trials because of its better operability and excellent effectiveness. CSS considers tumor mortality as the endpoint event and has better specificity in evaluating specific diseases. However, sampling and follow-up collection are more difficult in clinical trials, and the SEER database compensates for this difficulty. Previous studies have analyzed HCC based on SEER databases, which extensively include AFP factors. The conclusion revolves around the correlation between AFP positivity and adverse prognosis, tumor progression and metastasis, premature death, and other outcomes [8, 20–22]. However, few studies have been conducted in AFP-negative patients [9, 13, 14], and most of which did not involve treatment. Our study collected data from all AFP-negative patients in the SEER database covering various treatment options, including surgery, radiotherapy, and chemotherapy.

The overall conclusions drawn by the two groups were similar but with slight differences. Various sociological factors influence the occurrence and prognosis of HCC [23–25]. Our study showed that the CSS group was less affected by social factors and that the age and ethnicity factors included in the OS group were not statistically significant in the CSS group. It is worth mentioning that Chen et al. pointed out the impact of marital status on the prognosis of patients with HCC of TNM stage Ia in that positive marital status is an independent predictor of longer OS and reviewed previous relevant studies to support it [26]. The overlap between the target populations of the two studies was relatively low. Therefore, the impact of marital factors may require a clear understanding of the patients’ scope of application. Tumor burden factors play a prominent role in predicting tumor prognosis. Tumor size and grade are independent prognostic indicators of OS [13, 27, 28], and their impact on survival time is consistent with common sense. The difference between the two groups was relatively small. ANHC is more common in the early stages of the disease and has a better overall prognosis. Currently, the most beneficial treatment methods include surgery and radiofrequency ablation [8, 11, 23, 29]. Particularly for stage 0 disease or patients without surgical contraindications, resection and transplantation are the preferred treatment methods and have a better long-term prognosis than radiofrequency ablation [29–31]. Our results draw the same conclusion that the benefits of resection, especially liver transplantation, are significant. For example, a 60-year-old patient with stage a ANHC who undergoes resection can benefit from approximately 0.25 to 0.75 years of overall survival compared to refusing treatment; the five-year cancer-specific survival rate will increase from 0.3 to 0.8 in this scenario. Chemotherapy and radiotherapy have relatively little impact on the prognosis and survival time. The median survival period for symptomatic late-stage cases treated with systemic therapy is 1-1.5 years [23]. Considering that both are mostly used for patients with late-stage disease, the extension of survival time is relatively low. However, it is still an important treatment method for unresectable tumors.

Tumor TNM stage is an independent risk factor for OS in patients with HCC [28]. and it’s widely used in clinical evaluation in the therapy of various kinds of carcinoma. The DCA results indicated that the net benefit of the nomogram in prognostic evaluation was superior to that of TNM staging. And we analyzed the area under the curve (AUC) of ROC, which also indicates that our nomogram has better diagnostic efficiency than TNM staging. With improvements in surgical technologies and the development of new treatment modalities, the prognosis and overall survival of patients with HCC will gradually improve. Over time, the proportion of patients with HCC with lower AFP and earlier BCLC stage has increased, and overall survival has improved [32].

Improvements in overall survival increased the number of potential patients and led to significant improvements in systemic therapy management [32]. The cut-off point for the year of diagnosis in our study was 2009, which was the beginning of the advent of tyrosine kinase inhibitors (TKIs) led by sorafenib [33]. Targeted medicines play an important role in improving patient survival. The application of neoadjuvant or conversion therapy effectively reduced the tumor burden before surgery [34, 35]. It can also be administered after surgery or ablation to reduce the risk of tumor recurrence or progression [36].

Starting in 2015, immunotherapy was introduced at this stage, and patient survival after this time point improved [32]. For example, the IMbrave-150 study established the effectiveness of the classic treatment regimen of atezolizumab combined with bevacizumab, which can be used as the first-line treatment for most advanced HCC [37]. Considering that the application of systemic therapy depends on excellent liver function, there is room for its application in patients with ANHC [37, 38]. An important part of the ANHC patient population has BCLC stage B disease, which can also benefit from systemic therapy [32, 39]. Unfortunately, the data of this study were obtained from the SEER database, and the immunotherapy or targeted therapy status of relevant patients was not collected.

There are still shortcomings in this study. We did not obtain other widely used treatment methods for HCC, such as ablation and interventional surgery, and the prognostic evaluation of these treatment methods may not be comprehensive. The main population in the SEER database comprises Caucasians, and its applicability to other countries or regions may be limited. Third, although T staging is determined based on tumor size, number, and extent of tumor invasion, the SEER database lacks key data on portal vessel invasion status, residual liver function, and underlying liver disease, etc. Therefore, some clinically critical tumor-related variable information, such as Child-Pugh class and MELD score, may be missing in our model. In addition, due to the retrospective nature of the study, we were unable to control the treatment of all patients, and their treatment may be susceptible to differences in residential location and time. More importantly, our prognostic model incorporated treatments, which may limit the utility of our study. In the future, a prospective multi-center clinical study on patients with AFP-negative HCC is still needed to incorporate more clinical parameters and further clarify the impact of these parameters on prognosis.

## Conclusion

Our study demonstrated that age, ethnicity, tumor diameter, tumor grade, surgery, chemotherapy, and radiotherapy were independent prognostic factors for patients with ANHC. Surgical treatment has the most significant benefits among the various treatment methods. We designed a reliable nomogram targeting the ANHC population to evaluate prognosis. Our nomogram model shows good predictive performance for both OS and CSS, and can divide ANHC patients into subgroups with completely different prognosis.

### Electronic supplementary material

Below is the link to the electronic supplementary material.

Supplementary Material 1

Supplementary Material 2

Supplementary Material 3

Supplementary Material 4

Supplementary Material 5

Supplementary Material 6

Supplementary Material 7

Supplementary Material 8

Supplementary Material 9

Supplementary Material 10

Supplementary Material 11

## Data Availability

The data that support the findings of this study are available from SEER database (https://seer.cancer.gov/) and from the corresponding authors upon reasonable request.
