# Supplementary material for: Prediction of survival and analysis of prognostic factors for patients with AFP negative hepatocellular carcinoma: a population-based study
Source: BMC Gastroenterol. 2024 Mar 4;24:93. doi: 10.1186/s12876-024-03185-z (PMC10910698; doi:10.1186/s12876-024-03185-z)
Supplement: Supplementary file 1 — Supplementary Material 1 [file 12876_2024_3185_MOESM1_ESM.docx]

# Supplementary material

***Comparison of Nomogram Model and TNM Stage for OS Prediction Accuracy***

The DCA curve shows that in the training cohort, the nomogram predicting OS has more value than TNM (Supplementary Figure 3A,3B and 3C). The DCA of the validation cohort showed that the nomogram was also more reliable than traditional TNM staging (Supplementary Figure 4A, 4B and 4C). Time-dependent ROC curve analysis showed that the predictive efficacy of our nomogram model in predicting 1-year OS, 3-year OS and 5-year OS was 86.16(95% CI: 83.82-88.5), 85.44(95% CI: 83.35-87.52), 84.57(95% CI: 82.12-87.02), which was significantly higher than TNM staging (Supplementary Table 1). The above results show that our nomogram is superior to the existing TNM staging.

***Comparison of Nomogram Model and TNM Stage for CSS Prediction Accuracy***

We also constructed the DCA curve and ROC curve analysis under the condition of setting CSS as terminal event and get the identical result in this group. The nomogram predicting CSS has more value than TNM in both the training cohort (Supplementary Figure 3D, 3E and 3F) and the validation cohort (Supplementary Figure 4D, 4E and 4F). Time-dependent ROC curve analysis showed that the predictive efficacy of our nomogram model in predicting 1-year CSS, 3-year CSS and 5-year CSS was 82.40 (95% CI: 79.89-84.91), 82.82 (95% CI: 80.70-84.94), 82.85 (95% CI: 80.44-85.25), which was significantly higher than TNM staging (Supplementary Table 2).
