# Supplementary material for: Prediction of survival and analysis of prognostic factors for patients with AFP negative hepatocellular carcinoma: a population-based study
Source: BMC Gastroenterol. 2024 Mar 4;24:93. doi: 10.1186/s12876-024-03185-z (PMC10910698; doi:10.1186/s12876-024-03185-z)
Supplement: Supplementary file 4 — Supplementary Material 4 [file 12876_2024_3185_MOESM4_ESM.docx]

**Supplementary Figure 4** Decision curve analysis of nomograms and conventional models. On the basis of overall survival (A-C) and cancer-specific survival (D-F) for 1, 3, and 5 years of ANHC in validation cohort.
