# Supplementary material for: Prediction of survival and analysis of prognostic factors for patients with AFP negative hepatocellular carcinoma: a population-based study
Source: BMC Gastroenterol. 2024 Mar 4;24:93. doi: 10.1186/s12876-024-03185-z (PMC10910698; doi:10.1186/s12876-024-03185-z)
Supplement: Supplementary file 5 — Supplementary Material 5 [file 12876_2024_3185_MOESM5_ESM.docx]

**Supplementary Figure 1** Calibration curves for the purpose of developing a nomogram. It’s for predicting the overall survival (A-C) and cancer-specific survival (D-F) for 1, 3, and 5 years of ANHC in training cohort. The dashed line depicts the ideal curve that would exist if the expected result and the actual circumstance were identical. If the anticipated occurrence rate of the calibration curve is closer to the dashed line than the measured occurrence rate, the model’s prediction ability is more accurate. The x-axis depicts the expected survival of ANHC for this nomogram, whereas the y-axis depicts the actual survival.
