# Supplementary material for: Prediction of survival and analysis of prognostic factors for patients with AFP negative hepatocellular carcinoma: a population-based study
Source: BMC Gastroenterol. 2024 Mar 4;24:93. doi: 10.1186/s12876-024-03185-z (PMC10910698; doi:10.1186/s12876-024-03185-z)
Supplement: Supplementary file 7 — Supplementary Material 7 [file 12876_2024_3185_MOESM7_ESM.docx]

**Supplementary Figure 2** Calibration curves for the purpose of developing a nomogram. It’s for predicting the overall survival (A-C) and cancer-specific survival (D-F) for 1, 3, and 5 years of ANHC in validation cohort. The x-axis depicts the expected survival of ANHC for this nomogram, whereas the y-axis depicts the actual survival.
