# Supplementary figures and images for: Prediction of survival and analysis of prognostic factors for patients with AFP negative hepatocellular carcinoma: a population-based study
Source: BMC Gastroenterol. 2024 Mar 4;24:93. doi: 10.1186/s12876-024-03185-z (PMC10910698; doi:10.1186/s12876-024-03185-z)

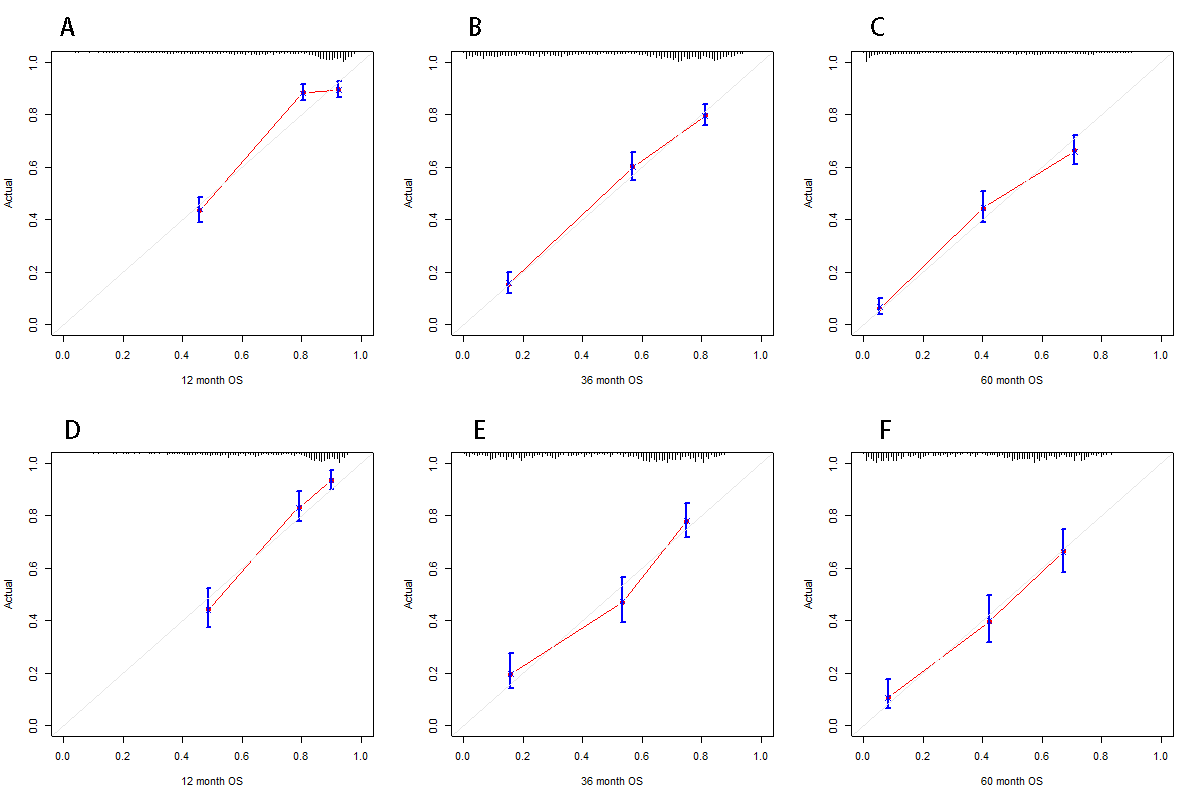

Supplement: Supplementary file 8 — Supplementary Material 8 [file 12876_2024_3185_MOESM8_ESM.png]

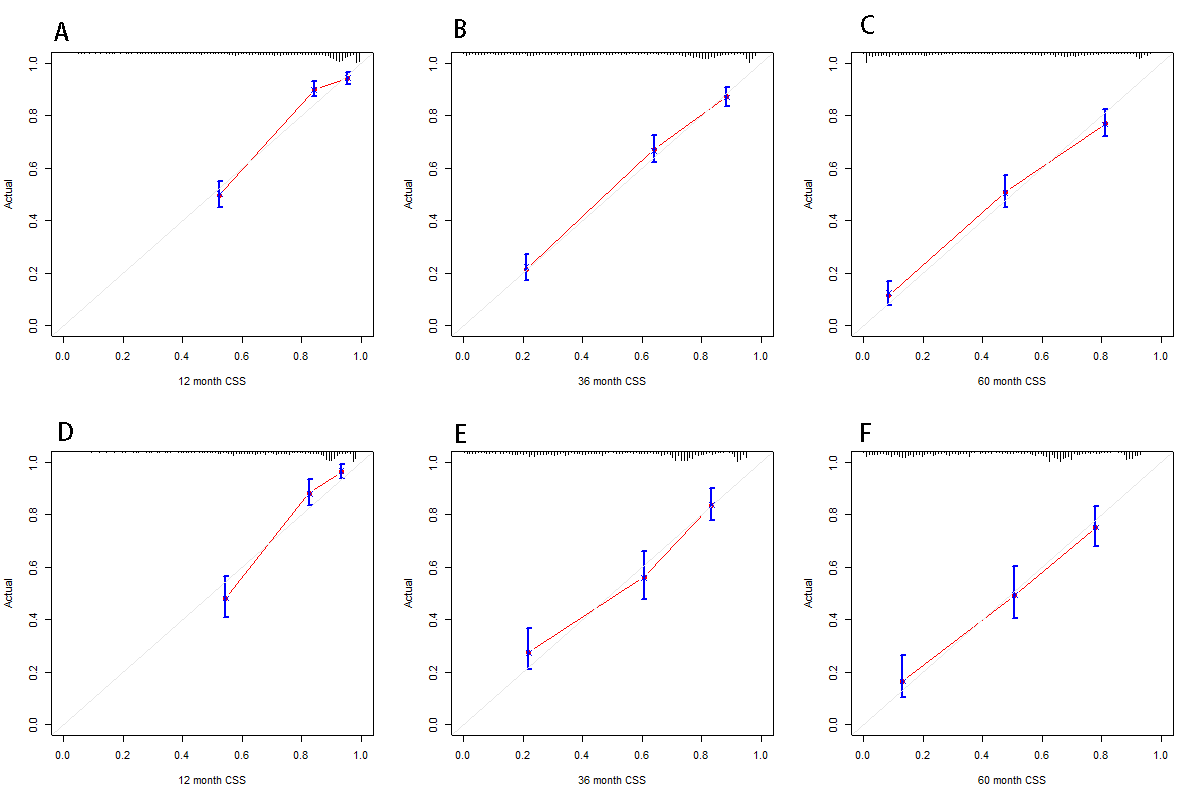

Supplement: Supplementary file 9 — Supplementary Material 9 [file 12876_2024_3185_MOESM9_ESM.png]

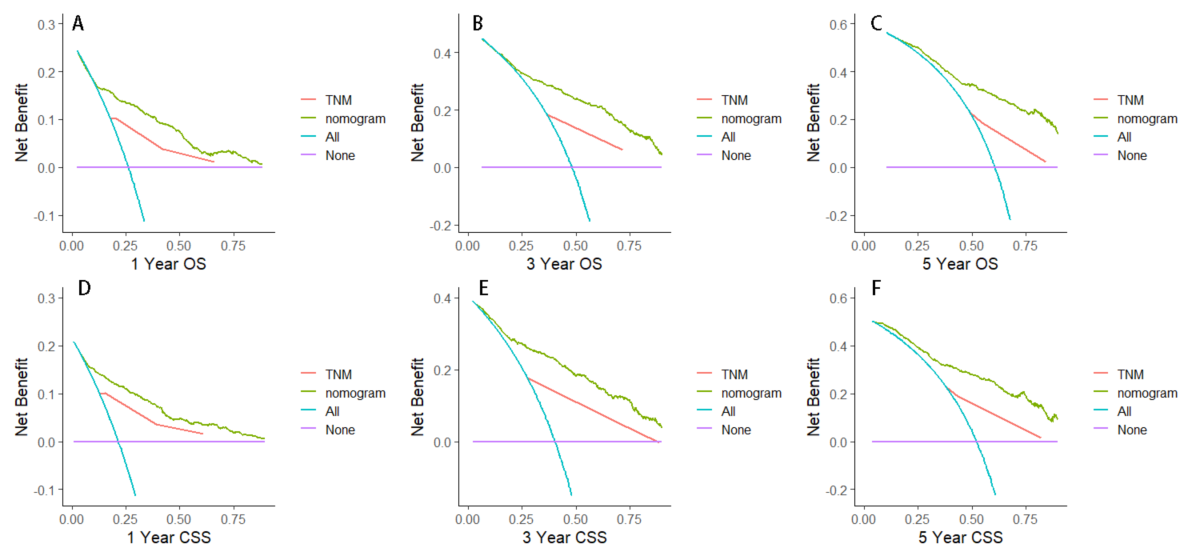

Supplement: Supplementary file 10 — Supplementary Material 10 [file 12876_2024_3185_MOESM10_ESM.png]

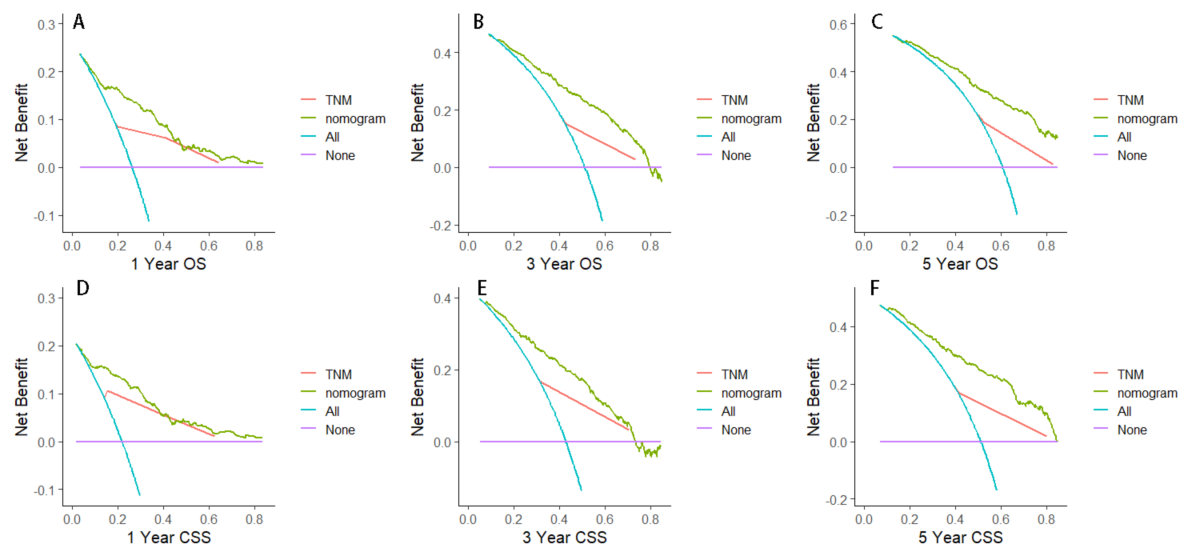

Supplement: Supplementary file 11 — Supplementary Material 11 [file 12876_2024_3185_MOESM11_ESM.png]
